# Cystic pelvi-abdominal mass in pregnancy: An uncommon presentation of a subserosal leiomyoma

**DOI:** 10.4102/sajr.v23i1.1683

**Published:** 2019-04-18

**Authors:** Natasha Sobey, Lauren Raubenheimer

**Affiliations:** 1 Department of Radiology, Inkosi Albert Luthuli Hospital, Durban, Kwa-Zulu Natal, South Africa; 2 Department of Radiology, Groote Schuur Hospital, University of Cape Town Medical School, Cape Town, South Africa

## Abstract

Uterine leiomyomas are common benign neoplasms. While generally easily diagnosed, variations in size and type of degeneration can drastically alter the imaging findings and result in diagnostic uncertainty. We present the case of a 34-year-old female with a large, cystic, pelvi-abdominal mass that was discovered in advanced pregnancy and misdiagnosed as an ovarian tumour. Intra-uterine demise of a viable foetus during an induction of labour and clinical deterioration necessitated further imaging and emergent surgical intervention. Further imaging, intra-operative findings and histopathological correlation revealed a pedunculated uterine leiomyoma with hyaline degeneration and areas of necrosis, complicated by left ovarian vein thrombosis. In addition to the intra-uterine demise, the unfortunate outcome included a total abdominal hysterectomy and unilateral salpingo-oophorectomy in a young patient. Typical leiomyomas are easily detected with imaging, however the varied clinical presentation and imaging findings in degenerating leiomyomas can prove a diagnostic challenge. This case describes a rare manifestation of a common condition and its subsequent diagnostic dilemma resulting in dire foeto-maternal outcomes.

**Keywords:**

Leiomyoma; Hypercalcaemia; Ovarian Vein Thrombosis; Pregnancy; Intra-Uterine Foetal Demise.

## Introduction

Uterine leiomyomas are a common benign neoplasm of the female pelvis, with a reported prevalence of 20%–40% in women of reproductive age.^1,2,3^ They are predominantly composed of smooth muscle cells with variable amounts of fibrous connective tissue.^2^

The current literature suggests that the majority of women with uterine fibroids will have normal pregnancy outcomes.^4^ No link was established between intra-uterine foetal demise and uterine leiomyomas in a meta-analysis in 2008.^5^ Subserosal leiomyomas in particular were also not found to be a significant cause of poor pregnancy outcome.^6^ However, the literature involving leiomyomas in pregnancy can be problematic to assess as many different variables have been described, including size, number, location, type and gestational age at detection. Simultaneous heterogeneity amongst the patient profile (age, parity, race and obesity) further complicates accurate analysis.^3^ As a result, the relationship between leiomyomas and adverse obstetric outcomes is incompletely understood.

Accurate diagnosis of leiomyomas in pregnancy is particularly important in a South African context as an increased prevalence of leiomyomas in the African population was described by Stewart et al.^3^ Local advances in reproductive health, as well as the current trend of delaying childbearing, further increase the relevance to the South African population, as treating clinicians can expect to see more women with leiomyomas presenting for antenatal and obstetric care.^5^

Typical leiomyomas are readily diagnosed radiologically; however, the appearance of uterine leiomyomas is commonly altered by degenerative changes including cystic, hyaline, myxoid and red degeneration. This can make differentiation between leiomyomas and other adnexal or other intra-abdominal pathology challenging.

Ultrasound is used to diagnose and monitor the growth of leiomyomas, and typically shows a well-defined hypoechoic mass. Intra-lesional cystic areas or calcification depends on the presence of complication or degeneration. Computed tomography (CT) generally depicts a soft tissue mass with variable enhancement, which distorts the uterine contour and may exhibit coarse peripheral or central calcification.

Typical magnetic resonance imaging (MRI) features of non-degenerated leiomyomas include a well-circumscribed mass of homogeneously low to intermediate signal intensity (as compared to myometrium) on T1-weighted images, decreased signal intensity on T2-weighted images with surrounding flow voids and variable contrast enhancement. Degenerated leiomyomas have variable but characteristic appearances depending on the type of degeneration. Defining features include high T1 signal intensity or an irregular, T1-hyperintense rim around a centrally located leiomyoma in red degeneration; high T2 signal intensity in cystic degeneration; low T2 signal intensity with poor enhancement in hyaline degeneration; and very high T2 signal intensity with gradual enhancement on T1-contrasted sequences in myxoid degeneration.^2^

In this case, a subserosal leiomyoma was misdiagnosed as an ovarian tumour antenatally. There are multiple reported cases of ovarian tumour-like leiomyomas^7^ but they are rarely reported in pregnancy.

## Case

A 34-year-old gravid female was referred to a tertiary hospital at 38+ weeks with a large cystic pelvi-abdominal (PA) mass detected on ultrasound. She had no known medical co-morbidities and one previous uneventful pregnancy culminating in a normal vaginal delivery (NVD) at full term. On presentation, the patient was asymptomatic and examination revealed only an increased symphysis-pubis fundal height (SFH) for expected gestation. Urine analysis showed proteinuria and blood tests revealed a minimally elevated cancer antigen-125 of 48 kU/L (normal range 0–35 kU/L) and a normal carcinoembryonic antigen level of 0.7 ng/mL (normal < 5.0 ng/mL).

Transabdominal ultrasound showed a thick-walled cystic mass measuring approximately 25 cm × 14 cm × 15 cm with no internal vascularity. The mass was assumed to be ovarian in origin. A singleton intra-uterine pregnancy with foetal heartbeat was confirmed. A transvaginal ultrasound scan was not performed.

**FIGURE 1:**
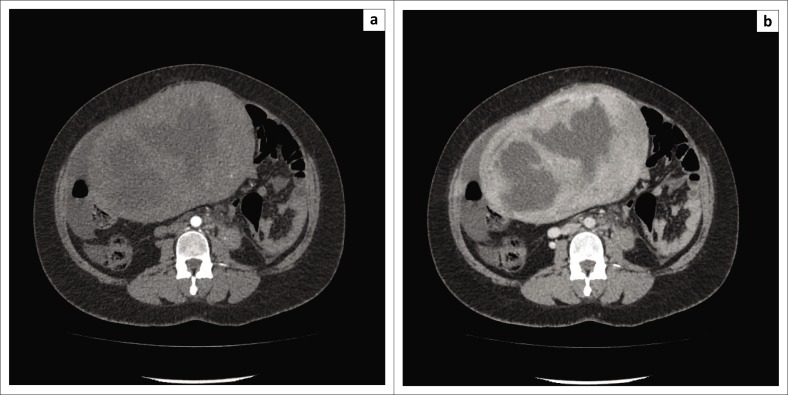
Axial computed tomography of the abdomen during (a) arterial and (b) portal venous phases showing a large centrally hypodense mid-abdominal mass with a thick enhancing wall and septations.

**FIGURE 2:**
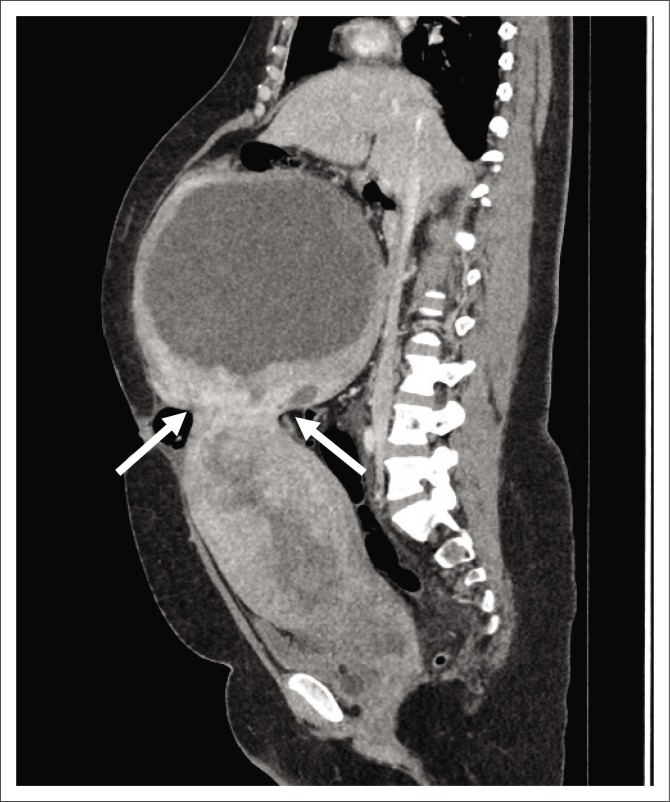
Sagittal portal venous computed tomography shows that the mass is connected to the uterine fundus (arrows).

**FIGURE 3:**
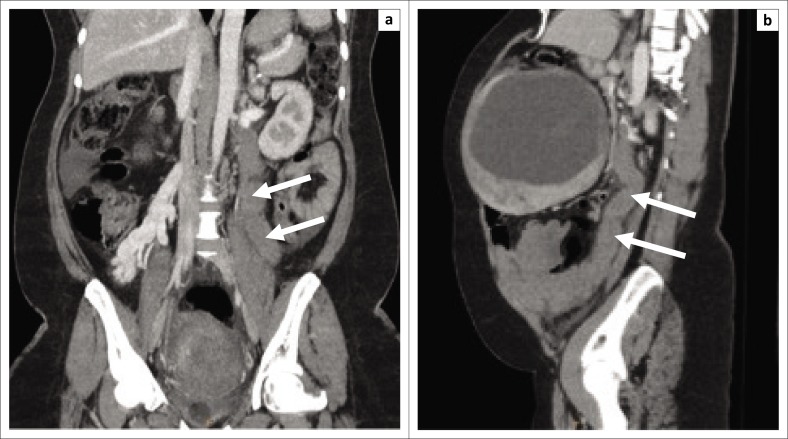
Coronal (a) and sagittal (b) portal venous computed tomography demonstrates tubular non-enhancing serpiginous structures corresponding to thrombosed left ovarian and parametrial veins (arrows).

**FIGURE 4:**
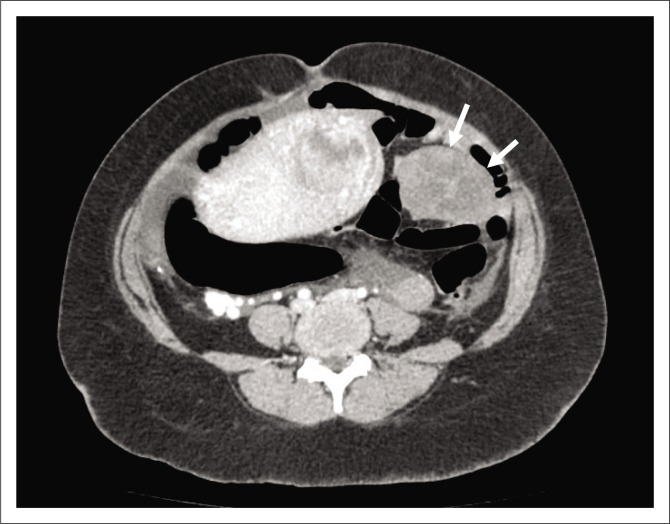
Poorly enhancing enlarged left ovary on axial portal venous computed tomography (arrows).

The decision was made to induce labour based on the unclassified proteinuria and the clinical suspicion of possible underlying pre-eclampsia. During the induction of labour, foetal monitoring showed subtle signs of foetal distress, which warranted continuous monitoring, but despite in-patient care and continuous foetal monitoring, there was subsequent intra-uterine foetal demise and the patient delivered a stillborn baby via NVD. Following delivery, the patient’s condition deteriorated in the ward, developing a persistent unexplained tachycardia of approximately 150 bpm, a blood pressure of 156/119 mmHg and a serum calcium level well above normal limits (4.25 mmol/L, normal limit is 2.62 mmol/L). Emergent cross-sectional imaging in the form of contrasted CT abdomen showed a large cystic mass with a thick, irregular enhancing wall and septations (Figure 1), which was inseparable from the uterine fundus (Figure 2). In addition, extensive left ovarian and pelvic vein thrombosis (Figure 3) and an enlarged, poorly enhancing left ovary were noted (Figure 4).

Exploratory laparotomy revealed a large, friable, thick-walled, pedunculated fundal mass, left ovarian vein thrombosis, necrosis of the left ovary and broad ligament and offensive intra-uterine contents. The decision to proceed to total abdominal hysterectomy and left salpingo-oophorectomy was taken based on significant intra-operative bleeding, friable necrotic tissue and haemodynamic instability. Histopathological evaluation revealed a subserosal uterine leiomyoma with extensive hyaline degeneration and necrosis, left ovarian vein thrombosis, haemorrhagic infarction of the left ovary and endometrial and myometrial necrosis.

## Ethical consideration

No patient identifiable information has been presented. Informed verbal consent was obtained from the patient prior to publication.

## Discussion

Leiomyomas are characterised by location, whether submucosal, intramural (within the myometrium) or subserosal. The latter are a subtype that may become pedunculated, projecting out of the uterine contour and mimicking an adnexal mass.^2^ Although usually asymptomatic, some pedunculated subserosal leiomyomas may undergo torsion, resulting in infarction. Rare but recognised complications linked to uterine leiomyomas in pregnancy include an increased risk of spontaneous abortion, foetal malpresentation, placenta previa, preterm birth, caesarean section and peripartum haemorrhage.^6^ Placental abruption is a rare but potentially devastating outcome that has been inconsistently associated with uterine leiomyomas.^6^ The strongest correlation with placental abruption has been documented in patients with submucosal or retroplacental leiomyomas.^5^

Ultrasound is employed as the primary diagnostic imaging modality because of its accessibility, safety and bedside application. Ultrasound gives information on leiomyoma size, characteristics and complications. Cross-sectional imaging is usually not required for initial diagnosis in typical cases and is reserved for patients with diagnostic uncertainty or suspected complications.

Coarse intra-lesional calcification, although an uncommon finding, remains the most specific sign for leiomyomas.^8^ Difficulties in detection can occur with smaller tumours, those that extend into the uterine cavity, those that occur only in the lower segment and in the absence of intra-lesional calcification. Internal heterogeneity depends on the type and degree of degeneration. A cystic appearance occurs with various forms of degeneration and is generally attributed to tumour growth beyond its vascular supply.^9^ Because of the varied imaging patterns, degenerating leiomyomas often pose a diagnostic challenge, as in the case of our patient. Hyaline degeneration is the most common type of degeneration, accounting for 60% of cases. Cystic degeneration is observed in 4% of leiomyomas and is the terminal sequelae of oedema.^10^

Confirming the diagnosis of ovarian vein thrombosis with ultrasound is highly operator dependent and may be confounded by patient factors such as overlying bowel gas and poor patient cooperation. Contrasted abdominal CT is sensitive and specific for ovarian vein thrombosis and should be considered as the initial investigation of choice in the absence of pregnancy. Computed tomography features include visualisation of a tubular retroperitoneal mass with central low attenuation extending cephalic to the inferior vena cava or left renal vein.^11^

Leiomyoma-associated hypercalcaemia is a rarely described entity, with seven reported cases to date.^12,13,14^ The presumed pathophysiology is leiomyoma-induced elevated serum parathyroid hormone-releasing protein (PTHrP), with a likely causal relationship proven in all but one case.^13^ In our patient’s case, serum PTHrP was not tested and the role of hypercalcaemia in the eventual outcome cannot be accurately extrapolated because of many concurrent variables. However, it should certainly encourage further research into PTHrP-secreting leiomyomas.

This case highlights the importance of employing multiple imaging modalities when confronted with a PA mass of uncertain origin in a pregnant patient. It also underscores the importance of a thorough search for the ovaries when imaging a PA mass, although this search may be hampered by distortion and displacement by large masses. Magnetic resonance imaging is the most accurate modality to evaluate tumour characteristics and may be utilised in the pregnant patient when ultrasonography is inconclusive.^2^

## Conclusion

Leiomyomas in pregnancy are common and are usually easily diagnosed, with no expected adverse obstetric complications. However, in rare cases, they may present in unpredictable ways with variable imaging findings that delay diagnosis and result in potentially harmful outcomes. Pedunculated uterine leiomyomas should be considered in the differential diagnosis of any PA mass. As degenerating leiomyomas are great mimics of other gynaecological and non-gynaecological medical conditions, familiarity with the different types of degeneration and their sonographic appearance is imperative to aid accurate diagnosis.

Ultrasound should be employed as the initial imaging modality of choice for PA masses in pregnancy. However, its limitations should be carefully considered and cross-sectional imaging should be used where appropriate.
